# Rhizosphere Engineering in Saline Soils: Role of PGPR and Organic Manures in Root–Soil Biochemical Interactions for Allium Crops

**DOI:** 10.3390/plants14193075

**Published:** 2025-10-04

**Authors:** Tarek Alshaal, Nevien Elhawat, Szilvia Veres

**Affiliations:** 1 Institute of Applied Plant Biology, Faculty of Agricultural and Food Sciences and Environmental Management, University of Debrecen, 4032 Debrecen, Hungaryszveres@agr.unideb.hu (S.V.); 2 Soil and Water Science Department, Faculty of Agriculture, Kafrelsheikh University, Kafr El-Sheikh 33516, Egypt; 3 Faculty of Agriculture (for Girls), Al-Azhar University, Nasr City 11884, Cairo, Egypt

**Keywords:** biofertilizers, organic manure, photosynthetic efficiency, oxidative stress, sustainable agriculture

## Abstract

Soil salinity disrupts rhizosphere interactions, impairing root–microbe symbioses, nutrient uptake, and water relations in onion (*Allium cepa* L.) and garlic (*Allium sativum* L.). This study evaluated the efficacy of biofertilizers (*Azotobacter chroococcum* SARS 10 and *Azospirillum lipoferum* SP2) and organic amendments (sewage sludge and poultry manure) in salt-affected soils in Kafr El-Sheikh, Egypt. Five treatments were applied: (T1) control (no amendments); (T2) biofertilizer (3 L/ha for onion, 12 L/ha for garlic) + inorganic P (150 kg/ha P_2_O_5_ for onion, 180 kg/ha for garlic) and K (115 kg/ha K_2_SO_4_ for onion, 150 kg/ha for garlic); (T3) 50% inorganic N (160 kg/ha for onion, 127.5 kg/ha for garlic) + 50% organic manure (6000 kg/ha for onion, 8438 kg/ha for garlic) + P and K; (T4) biofertilizer + T3; and (T5) conventional inorganic NPK (320 kg/ha N for onion, 255 kg/ha N for garlic + P and K). Soil nutrients (N, P, K), microbial biomass carbon (MBC), dehydrogenase activity, and microbial populations were analyzed using standard protocols. Plant growth (chlorophyll, photosynthetic rate), stress indicators (malondialdehyde, proline), and yield (bulb diameter, fresh yield) were measured. Treatment T4 increased MBC by 30–40%, dehydrogenase activity by 25–35%, available N (39.7 mg/kg for onion, 35.7 mg/kg for garlic), P (17.9 mg/kg for onion), and K (108 mg/kg for garlic). Soil organic matter rose by 8–12%, and cation exchange capacity by 26–36%. Chlorophyll content improved by 25%, malondialdehyde decreased by 20–30%, and fresh yields increased by 20–30% (12.17 tons/ha for garlic). A soybean bioassay confirmed sustained fertility with 20–25% higher dry weight and 30% greater N uptake in T4 plots. These findings highlight biofertilizers and organic amendments as sustainable solutions for *Allium* productivity in saline rhizospheres.

## 1. Introduction

Soil salinity poses a significant environmental challenge to global agricultural productivity. It adversely affects plant growth and yield by inducing osmotic stress, ion toxicity, and nutrient imbalances [1]. Onion (*Allium cepa* L.) and garlic (*Allium sativum* L.) are particularly sensitive to saline conditions, which lead to reduced germination rates, stunted plant growth, decreased bulb weight, and substantial yield losses [2]. Elevated salt concentrations impair water uptake, causing physiological drought and triggering oxidative stress through the accumulation of reactive oxygen species (ROS) [3]. These ROS damage cellular components, compromising crop quality and shelf life [4]. Additionally, excessive sodium (Na^+^) and chloride (Cl^-^) ions disrupt the uptake of essential nutrients like potassium (K^+^) and calcium (Ca^2+^), which are vital for cell expansion and membrane stability [5]. Implementing sustainable agronomic practices is critical to mitigate salinity-induced stress and enhance onion and garlic production in affected regions.

Onion and garlic are among the most widely cultivated vegetable crops worldwide, valued for their nutritional, medicinal, and economic contributions. They are rich in bioactive compounds, vitamins, and minerals that support human health [6]. Onions contain sulfur-containing compounds, such as quercetin and flavonoids, which exhibit antioxidant, anti-inflammatory, and antimicrobial properties [7]. Garlic is renowned for its organosulfur compounds, particularly allicin, which contribute to its ability to lower cholesterol, regulate blood pressure, and enhance immune function [8]. These health benefits make both crops essential dietary staples globally. Economically, onion and garlic are high-value crops that play a pivotal role in global trade and farmer livelihoods. They are cultivated extensively across diverse regions, contributing significantly to agricultural economies. For instance, onions are a major commodity in international markets, with global production exceeding 100 million tonnes annually, driven by demand in both fresh and processed forms [9]. Garlic, similarly, is a key crop in countries like China, India, and the United States, supporting rural economies through domestic consumption and exports [10]. These crops are critical for food security, providing affordable nutrition and generating income for smallholder farmers. Enhancing their productivity through effective fertilization and salinity management strategies is essential to meet rising market demands and sustain agricultural economies.

Organic nitrogen sources, including poultry manure and sewage sludge, provide a sustainable alternative to inorganic nitrogen fertilizers. These amendments improve soil fertility by enhancing organic matter content, microbial activity, and nutrient availability [11]. Poultry manure is rich in nitrogen, phosphorus, and potassium, promoting vigorous plant growth and higher crop yields [12]. Sewage sludge, when properly treated, provides essential macronutrients and micronutrients while improving soil structure and water retention capacity [13]. Studies have demonstrated that organic amendments enhance root development, nutrient uptake, and stress tolerance in salt-affected soils [14]. Additionally, long-term application of organic fertilizers reduces environmental pollution and improves soil carbon sequestration [7]. However, appropriate management practices are necessary to mitigate potential risks associated with heavy metal accumulation in soils amended with sewage sludge [15].

Biofertilizers containing beneficial microorganisms such as *Azotobacter* and *Azospirillum* offer a promising approach for reducing reliance on inorganic nitrogen fertilizers. These nitrogen-fixing bacteria enhance soil fertility by converting atmospheric nitrogen into plant-available forms, reducing the need for synthetic fertilizers [16,17,18]. *Azotobacter* species produce phytohormones that stimulate root elongation, enhance nutrient uptake, and improve stress resistance in crops [19]. *Azospirillum* inoculation promotes plant growth by facilitating nitrogen fixation and enhancing water-use efficiency, making it particularly beneficial for crops grown under saline conditions [20]. Biofertilizers also improve soil microbial diversity and suppress soil-borne pathogens, further contributing to sustainable agricultural practices [21]. Integrating biofertilizers with organic amendments can optimize nutrient availability and soil health, leading to improved onion and garlic production.

A combined approach using organic nitrogen sources and biofertilizers provides a balanced nutrient supply while enhancing soil biological activity and sustainability. This strategy reduces dependence on synthetic fertilizers, which are associated with soil degradation and greenhouse gas emissions [22]. Studies have shown that integrating poultry manure and biofertilizers significantly improves soil organic carbon levels, microbial biomass, and enzymatic activity, leading to better nutrient cycling and plant growth [23]. The synergistic effect of biofertilizers and organic amendments enhances root development, water retention, and stress tolerance in salt-affected soils, resulting in higher crop yields and improved bulb quality in onions and garlic [24]. Furthermore, this approach aligns with sustainable agriculture goals by promoting environmentally friendly practices and reducing the negative impacts of excessive inorganic fertilizer use [25].

Soil salinity, characterized by high sodium (Na^+^) levels (e.g., initial EC_e_: 3.47 dS/m), induces osmotic stress, ion toxicity, and nutrient imbalances, reducing onion and garlic productivity [1,2]. This study aims to evaluate fertilization strategies integrating biofertilizers (*Azotobacter chroococcum* SARS 10, *Azospirillum lipoferum* SP2) and organic amendments (poultry manure, sewage sludge) to enhance soil fertility, plant growth, and yield quality in salt-affected soils. Although amendments may slightly increase soil ECe, their ability to improve soil organic matter (SOM), cation exchange capacity (CEC), and microbial activity, while enhancing plant stress tolerance through phytohormone production and K^+^/Na^+^ selectivity, makes them effective in mitigating salinity stress. This approach reduces reliance on inorganic fertilizers, promoting sustainable productivity in high-salinity environments.

## 2. Results

### 2.1. Soil Fundamental Properties

Soil properties varied across five treatments (T1–T5) for onion and garlic crops, with differences in pH, salinity, fertility, and nutrient levels (Table 1). The control (T1) had a pH of 8.20 for both crops. Treatments T2–T5 showed slight pH changes (7.90–8.30 for onion, 8.00–8.10 for garlic), indicating minimal impact from amendments. Salinity, measured by electrical conductivity (EC_e_), was lowest in T1 (4.00 for onion, 3.84 for garlic) and highest in T5 (4.71 for onion, 4.99 for garlic), with T3 and T4 also showing elevated levels. Soil organic matter (SOM) and cation exchange capacity (CEC) were lowest in T1 (SOM: 22.8 for onion, 22.2 for garlic; CEC: 34.4 for onion, 34.1 for garlic) and highest in T3 and T4 (SOM: 24.3–24.7 for onion, 23.4–24.8 for garlic; CEC: 43.5–46.7). The Soil Fertility Index (SFI) was lowest in T1 (0.94) and highest in T4 (1.00), with T5 slightly lower (0.98–0.99).

Nutrient levels varied significantly. Available nitrogen (Ava-N) was lowest in T1 (27.3 mg/kg for onion, 28.0 mg/kg for garlic) and highest in T3 and T4 (37.4–39.7 mg/kg for onion, 35.4–35.7 mg/kg for garlic). Available phosphorus (Ava-P) followed a similar trend, lowest in T1 (8.5 mg/kg for onion, 9.5 mg/kg for garlic) and highest in T3 and T4 (15.4–17.9 mg/kg for onion, 13.0–13.6 mg/kg for garlic). Available potassium (Ava-K) was lowest in T1 (91 mg/kg for onion, 95 mg/kg for garlic) and highest in T3 and T4 (105–108 mg/kg). Total nitrogen (Total-N) ranged from 1.22–1.23 g/kg in T1 to 1.45–1.51 g/kg in T3 and T4. Total phosphorus (Total-P) was stable across treatments (0.22–0.26 g/kg), showing minimal amendment impact.

Heavy metal levels (lead, nickel, copper) remained within safe agricultural limits. Available lead (Ava-Pb) was lowest in T1 (0.61 mg/kg) and highest in T5 (0.66–0.72 mg/kg). Available nickel (Ava-Ni) ranged from 0.38–0.39 mg/kg in T1 to 0.42–0.48 mg/kg in T5. Available copper (Ava-Cu) was lowest in T1 (1.33 mg/kg) and highest in T5 (1.84–2.10 mg/kg). Organic amendments in T3, T4, and T5 increased metal availability but stayed safe for soil and crop health.

Treatments T3 and T4 consistently showed the highest fertility, nutrient availability, and organic matter, likely due to organic manure and biofertilizer use. Salinity increased with amendments, especially in T5. Heavy metal levels, while elevated in amended soils, remained safe. Amendments had minimal impact on pH and total phosphorus.

### 2.2. Soil Microbial Parameters

**Figure 1:**
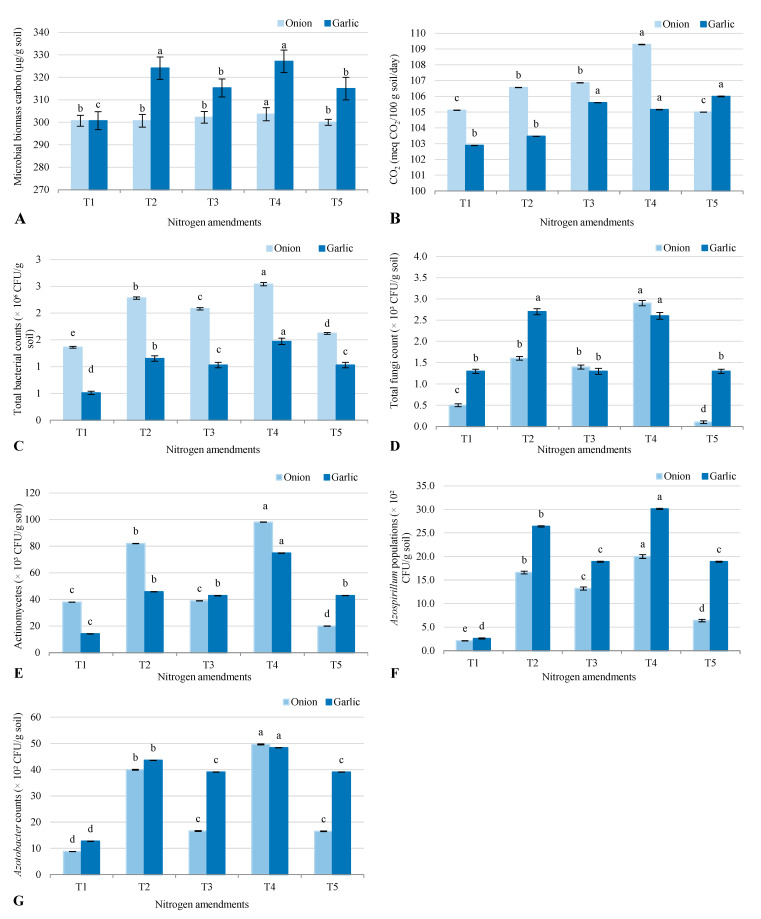
Response of soil microbial biomass carbon (MBC) (**A**); soil respiration (CO_2_) (**B**); total microbial counts (**C**–**G**) to different nitrogen amendments (T1–T5) under onion (*Allium cepa* L.) and garlic (*Allium sativum* L.) cultivations in salt-affected soil. T1: Control (no fertilizers or amendments), T2: Biofertilizer inoculation (*Azotobacter chroococcum* SARS 10 and *Azospirillum lipoferum* SP2) + recommended inorganic P and K, T3: 50% inorganic N and 50% organic manure (sewage sludge and poultry manure in a 1:1 ratio based on N content) + recommended P and K, T4: Biofertilizer + T3, and T5: Conventional inorganic NPK fertilizers. The recommended inorganic NPK were calculated per hectare per each crop (onion and garlic) according to the recommendations from the Ministry of Agriculture and Soil Reclamation, Egypt. Different letters on bars are significant according to Tukey’s test at *p* ≤ 0.05. Data are means ± SD. (*n* = 3).

The results show significant differences in microbial biomass carbon (MBC), soil CO_2_ emissions, and microbial populations across treatments in salt-affected soil after growing onion and garlic (Figure 1). MBC remained relatively stable across treatments. The control (T1) had the lowest MBC (301 mg/kg for both crops). Slight increases were observed in T2 (324 mg/kg for garlic) and T4 (327 mg/kg for garlic), with T3 and T5 showing intermediate values. This suggests that biofertilizer application in T2 and T4 slightly boosted microbial biomass. Soil CO_2_, an indicator of microbial activity, varied slightly across treatments. T4 showed the highest levels (109 mg/kg for onion, 105 mg/kg for garlic), reflecting enhanced microbial activity likely due to combined biofertilizer and organic manure. T2 and T3 had moderate CO_2_ levels, while T1 and T5 recorded the lowest, indicating lower microbial activity in these treatments. Bacterial counts were lowest in T1 (1.36 × 10^6^ CFU/g for onion, 0.51 × 10^6^ CFU/g for garlic) and highest in T4 (2.54 × 10^6^ CFU/g for onion, 1.47 × 10^6^ CFU/g for garlic), showing that biofertilizers and organic amendments in T4 significantly increased bacterial populations. Fungal counts followed a similar trend, peaking in T4 (2.9 × 10^6^ CFU/g for onion, 2.6 × 10^6^ CFU/g for garlic) but dropping to the lowest in T5 for onion (0.1 × 10^6^ CFU/g), suggesting that inorganic fertilizers in T5 may suppress fungal growth. Actinomycetes populations were also highest in T4 (98.0 × 10^3^ CFU/g for onion, 74.8 × 10^3^ CFU/g for garlic), while T1 and T5 had lower counts. Nitrogen-fixing bacteria, *Azotobacter* and *Azospirillum*, thrived in T4 (49.7 × 10^3^ CFU/g and 48.4 × 10^3^ CFU/g for onion and garlic, respectively), highlighting the effectiveness of biofertilizers in promoting these beneficial microbes.

**Figure 2:**
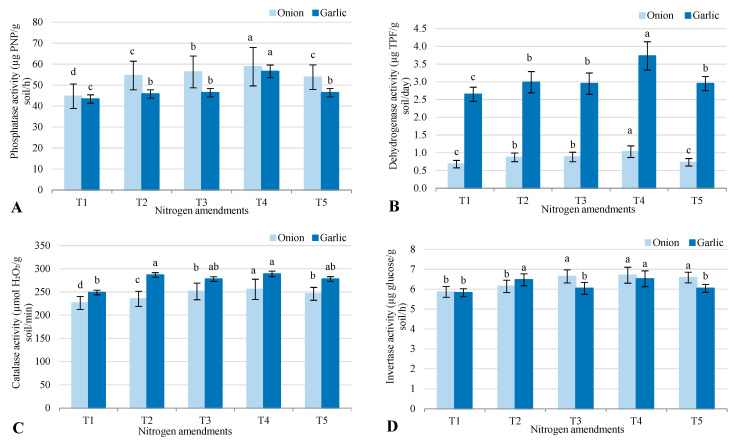
Response of soil enzyme activities (**A**) phosphatase; (**B**) dehydrogenase; (**C**) catalase; and (**D**) invertase to different agronomic practices (T1–T5) under onion (*Allium cepa* L.) and garlic (*Allium sativum* L.) cultivations in salt-affected soil. T1: Control (no fertilizers or amendments), T2: Biofertilizer inoculation (*Azotobacter chroococcum* SARS 10 and *Azospirillum lipoferum* SP2) + recommended inorganic P and K, T3: 50% inorganic N and 50% organic manure (sewage sludge and poultry manure in a 1:1 ratio based on N content) + recommended P and K, T4: Biofertilizer + T3, and T5: Conventional inorganic NPK fertilizers. The recommended inorganic NPK were calculated per hectare per each crop (onion and garlic) according to the recommendations from the Ministry of Agriculture and Soil Reclamation, Egypt. Different letters on bars are significant according to Tukey’s test at *p* ≤ 0.05. Data are means ± SD. (*n* = 3).

Soil enzyme activities varied across treatments in salt-affected soil after cultivating onion and garlic, with notable differences in phosphatase, dehydrogenase, catalase, and invertase activities (Figure 2). Phosphatase activity was consistently higher in onion than in garlic across all treatments. The highest levels were recorded in T4 (58.8 for onion, 56.6 for garlic), while the lowest were in T1 (44.7 for onion, 43.4 for garlic). This indicates that the combination of biofertilizer with organic and inorganic amendments in T4 significantly boosted phosphatase activity, particularly for onion. Dehydrogenase activity showed the opposite trend, with garlic consistently higher than onion. The highest values were in T4 (3.73 for garlic, 1.03 for onion), while T1 had the lowest (0.68 for onion, 2.65 for garlic). This suggests that garlic cultivation, especially under biofertilizer and organic amendments (T4), strongly enhanced dehydrogenase activity. Catalase activity was also higher in garlic than in onion. T4 showed the highest levels for both crops (289 for garlic, 256 for onion), while T1 had the lowest (249 for garlic, 226 for onion). The combination of biofertilizer and organic–inorganic amendments in T4 was most effective in increasing catalase activity, particularly for garlic. Invertase activity showed smaller differences between onion and garlic. The highest activity for onion was in T4 (6.70), while for garlic, it was in T2 (6.47). T1 recorded the lowest values (5.86 for onion, 5.82 for garlic). This suggests invertase activity was less affected by treatment type, though T4 still enhanced it for onion, reinforcing its role in improving soil enzyme activity.

### 2.3. Plant Parameters

**Figure 3:**
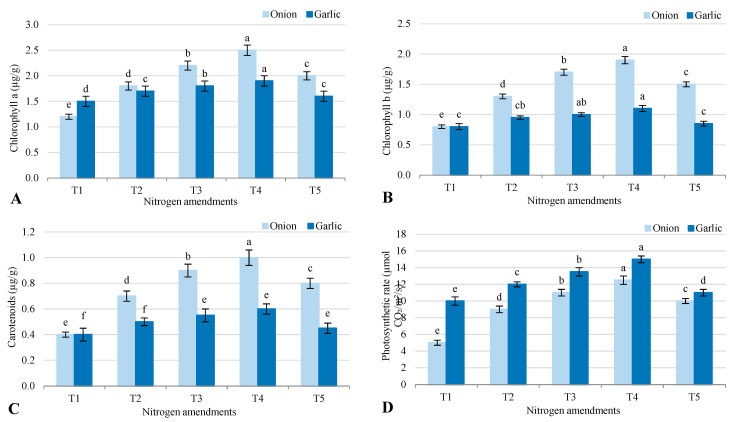
Response of photosynthetic machinery (**A**) chlorophyll a; (**B**) chlorophyll a; (**C**) carotenoids; and (**D**) photosynthetic rate to different agronomic practices (T1–T5) under onion (*Allium cepa* L.) and garlic (*Allium sativum* L.) cultivations in salt-affected soil. T1: Control (no fertilizers or amendments), T2: Biofertilizer inoculation (*Azotobacter chroococcum* SARS 10 and *Azospirillum lipoferum* SP2) + recommended inorganic P and K, T3: 50% inorganic N and 50% organic manure (sewage sludge and poultry manure in a 1:1 ratio based on N content) + recommended P and K, T4: Biofertilizer + T3, and T5: Conventional inorganic NPK fertilizers. The recommended inorganic NPK were calculated per hectare per each crop (onion and garlic) according to the recommendations from the Ministry of Agriculture and Soil Reclamation, Egypt. Different letters on bars are significant according to Tukey’s test at *p* ≤ 0.05. Data are means ± SD. (*n* = 3).

Photosynthetic pigments and rates in onion and garlic grown in salt-affected soil varied significantly across treatments (Figure 3). Chlorophyll a levels were lowest in the control (T1) at 1.2 mg/g for onion and 1.5 mg/g for garlic, while T4 (biofertilizer + organic manure) showed the highest levels at 2.5 mg/g for onion and 1.9 mg/g for garlic. T2 (biofertilizer) increased levels to 1.8 mg/g for onion and 1.7 mg/g for garlic, and T5 (inorganic fertilizers) had moderate levels at 2.0 mg/g for onion and 1.6 mg/g for garlic. Chlorophyll b followed a similar pattern, with T1 lowest at 0.8 mg/g for both crops, T4 highest at 1.9 mg/g for onion and 1.1 mg/g for garlic, and T3 and T5 showing intermediate values. Carotenoid levels were also lowest in T1 (0.4 mg/g for both crops) and highest in T4 (1.0 mg/g for onion, 0.6 mg/g for garlic), with T3 outperforming T5. Photosynthetic rates were lowest in T1 (5.0 µmol CO_2_/m^2^/s for onion, 10 for garlic) and highest in T4 (12.5 for onion, 15 for garlic), followed by T3 (11.0 for onion, 13.5 for garlic), while T5 had lower rates (10 for onion, 11 for garlic). Overall, biofertilizer and organic manure in T3 and T4 consistently enhanced pigment levels and photosynthetic rates compared to inorganic fertilizers in T5 or the control.

**Figure 4:**
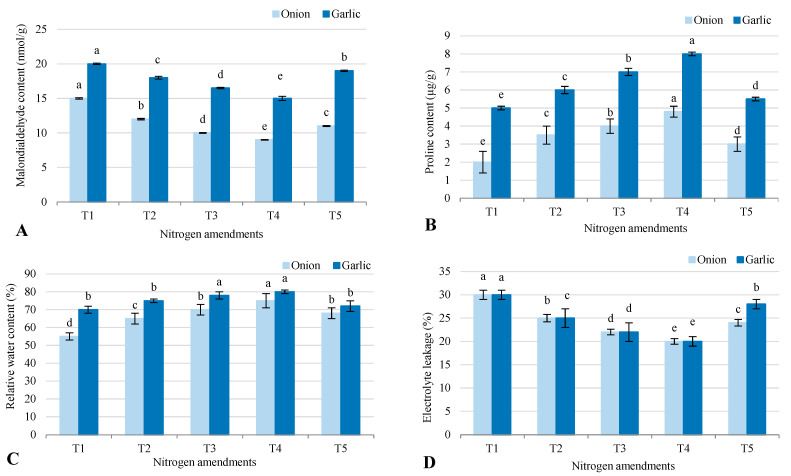
Response of different stress indicators (**A**) malondialdehyde content; (**B**) proline content; (**C**) relative water content; and (**D**) electrolyte leakage to different agronomic practices (T1–T5) under onion (*Allium cepa* L.) and garlic (*Allium sativum* L.) cultivations in salt-affected soil. T1: Control (no fertilizers or amendments), T2: Biofertilizer inoculation (*Azotobacter chroococcum* SARS 10 and *Azospirillum lipoferum* SP2) + recommended inorganic P and K, T3: 50% inorganic N and 50% organic manure (sewage sludge and poultry manure in a 1:1 ratio based on N content) + recommended P and K, T4: Biofertilizer + T3, and T5: Conventional inorganic NPK fertilizers. The recommended inorganic NPK were calculated per hectare per each crop (onion and garlic) according to the recommendations from the Ministry of Agriculture and Soil Reclamation, Egypt. Different letters on bars are significant according to Tukey’s test at *p* ≤ 0.05. Data are means ± SD. (*n* = 3).

In onion and garlic grown in salt-affected soil, oxidative stress, osmoprotectant levels, hydration, and cell membrane stability varied significantly across treatments (Figure 4). Malondialdehyde (MDA), an indicator of oxidative stress, was higher in garlic than onion, with the highest levels in the control (T1) at 20 nmol/g for garlic and 15 nmol/g for onion, and the lowest in T4 (9 nmol/g for onion, 15 nmol/g for garlic), where biofertilizer combined with organic and inorganic amendments reduced oxidative damage. Proline, an osmoprotectant aiding stress tolerance, was also higher in garlic, with T4 showing the highest levels (8 µmol/g for garlic, 4.8 µmol/g for onion) and T1 the lowest (5 µmol/g for garlic, 2.0 µmol/g for onion), indicating T4’s role in enhancing stress resilience. Relative water content (RWC), a measure of hydration, was highest in T4 (80% for garlic, 75% for onion) and lowest in T1 (70% for garlic, 55% for onion), suggesting T4 improved water retention under saline conditions. Electrolyte leakage, reflecting cell membrane stability, was highest in T1 (30% for both crops), indicating greater membrane damage, while T4 had the lowest (20% for both), showing better membrane integrity and reduced stress-induced damage. Overall, T4 was most effective in mitigating oxidative stress, boosting proline, improving hydration, and preserving membrane stability in both crops.

**Figure 5:**
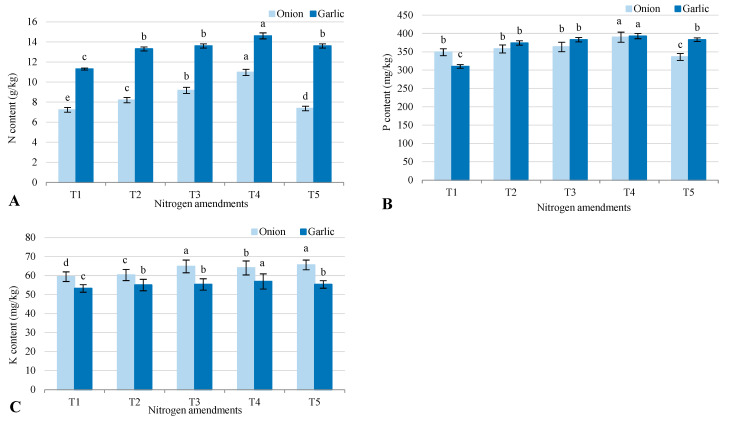
Response of plant NPK (**A**) N content; (**B**) P content; and (**C**) K content to different nitrogen amendments (T1–T5) under onion (*Allium cepa* L.) and garlic (*Allium sativum* L.) cultivations in salt-affected soil. T1: Control (no fertilizers or amendments), T2: Biofertilizer inoculation (*Azotobacter chroococcum* SARS 10 and *Azospirillum lipoferum* SP2) + recommended inorganic P and K, T3: 50% inorganic N and 50% organic manure (sewage sludge and poultry manure in a 1:1 ratio based on N content) + recommended P and K, T4: Biofertilizer + T3, and T5: Conventional inorganic NPK fertilizers. The recommended inorganic NPK were calculated per hectare per each crop (onion and garlic) according to the recommendations from the Ministry of Agriculture and Soil Reclamation, Egypt. Different letters on bars are significant according to Tukey’s test at *p* ≤ 0.05. Data are means ± SD. (*n* = 3).

Nutrient levels (nitrogen, phosphorus, and potassium) in onion and garlic grown in salt-affected soil varied significantly across treatments (Figure 5). Nitrogen content was higher in garlic than onion, with T4 (biofertilizer + organic–inorganic amendments) showing the highest levels (14.6 for garlic, 10.97 for onion) and the control (T1) the lowest (11.3 for garlic, 7.23 for onion), indicating T4’s effectiveness in boosting nitrogen uptake, particularly in garlic. Phosphorus content was generally higher in onion than garlic, except in T2 and T3, where garlic had a slight advantage. T4 had the highest phosphorus levels (390 for onion, 393 for garlic), while T1 had the lowest (349 for onion, 310 for garlic), showing T4’s strong impact on phosphorus availability, especially for onion. Potassium levels were higher in onion than garlic across all treatments. For onion, T5 (inorganic NPK fertilizers) had the highest potassium content (65.6), while for garlic, T4 was highest (56.9). T1 showed the lowest potassium levels (59.4 for onion, 53.2 for garlic), underscoring the importance of fertilization for nutrient uptake. Overall, T4 enhanced nitrogen and phosphorus uptake, while T5 was most effective for potassium in onion, and T4 for potassium in garlic.

The field experiment showed that different treatments significantly affected the yield and yield components of onion and garlic in salt-affected soil (Table 2). Treatment T4 (biofertilizer + 50% inorganic nitrogen + 50% organic manure) consistently outperformed others, producing the highest fresh mass (FM) with leaves (105.3 g/plant for onion, 92.1 g/plant for garlic) and without leaves (76.3 g/plant for onion, 62.8 g/plant for garlic), dry mass (DM) with leaves (11.3 g/plant for onion, 23.1 g/plant for garlic) and without leaves (7.2 g/plant for onion, 15.4 g/plant for garlic), bulb diameter (5.2 cm for onion, 5.0 cm for garlic), fresh yield (11.78 tons/ha for onion, 12.17 tons/ha for garlic), and dry yield (11.06 tons/ha for onion, 10.17 tons/ha for garlic). T4 also achieved the highest crop response factor (CRF) values (20.1 for onion, 11.5 for garlic), indicating efficient nutrient and water use. Treatment T3 (50% inorganic nitrogen + 50% organic manure) improved yields over the control (T1), but was less effective than T4. Treatment T5 (conventional inorganic fertilizers) performed similarly to T3 but was outperformed by T4. Treatment T2 (biofertilizer alone) showed moderate improvements over T1 but was less effective than T3 and T4. The control (T1) had the lowest performance across all parameters, highlighting the critical role of fertilization strategies in enhancing crop production in salt-affected soils.

### 2.4. Residual Effects

**Figure 6:**
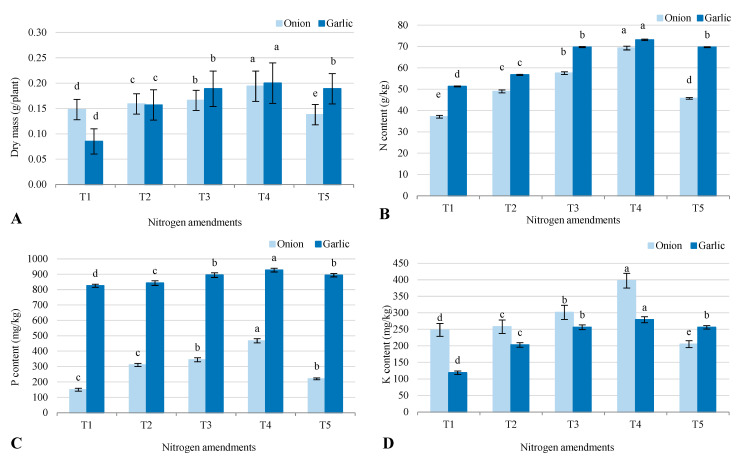
Response of soybean plants grown for 21 days, (**A**) dry mass; (**B**) N content; (**C**) P content; and (**D**) K content, as a model for studying the residual effect of five different nitrogen amendments (T1–T5) under onion (*Allium cepa* L.) and garlic (*Allium sativum* L) cultivation in salt-affected soil. T1: Control (no fertilizers or amendments), T2: Biofertilizer inoculation (*Azotobacter chroococcum* SARS 10 and *Azospirillum lipoferum* SP2) + recommended inorganic P and K, T3: 50% inorganic N and 50% organic manure (sewage sludge and poultry manure in a 1:1 ratio based on N content) + recommended P and K, T4: Biofertilizer + T3, and T5: Conventional inorganic NPK fertilizers. The recommended inorganic NPK were calculated per hectare per each crop (onion and garlic) according to the recommendations from the Ministry of Agriculture and Soil Reclamation, Egypt. Different letters on bars are significant according to Tukey’s test at *p* ≤ 0.05. Data are means ± SD. (*n* = 3).

The field experiment showed that different treatments significantly affected the dry weight and nutrient content (nitrogen, phosphorus, and potassium) of soybean plants grown in soil previously used for onion and garlic under salt-affected conditions (Figure 6). Treatment T4 (biofertilizer combined with 50% inorganic nitrogen and 50% organic manure) produced the highest soybean dry weight in onion (0.194 g) and garlic (0.200 g) plots, indicating improved soil fertility and plant growth. T4 also showed the highest nitrogen (N) content (69.3 mg/kg for onion plots, 73.1 mg/kg for garlic plots), phosphorus (P) content (467 mg/kg for onion, 927 mg/kg for garlic), and potassium (K) content (397 mg/kg for onion, 279 mg/kg for garlic). This highlights T4’s superior nutrient availability and uptake efficiency. T3 (50% inorganic nitrogen and 50% organic manure) performed well, with notable increases in soybean dry weight and N, P, and K content compared to the control (T1), though it was slightly less effective than T4. T5 (conventional inorganic fertilizers) showed moderate results, with N, P, and K levels similar to T3 but lower than T4, suggesting that biofertilizers combined with organic amendments enhanced nutrient use efficiency. T2 (biofertilizer alone) provided moderate improvements over T1 but was less effective than T3 and T4. The control (T1) had the lowest values for all parameters, underscoring the importance of fertilization for improving soil fertility and crop productivity in salt-affected soils. Soybean grown in garlic plots generally had higher nutrient content, especially phosphorus, compared to onion plots. This suggests a residual effect of treatments on soil nutrient dynamics, particularly from garlic cultivation.

### 2.5. Soil–Plant Interactions (Pearson Correlation and PCA)

**Figure 7:**
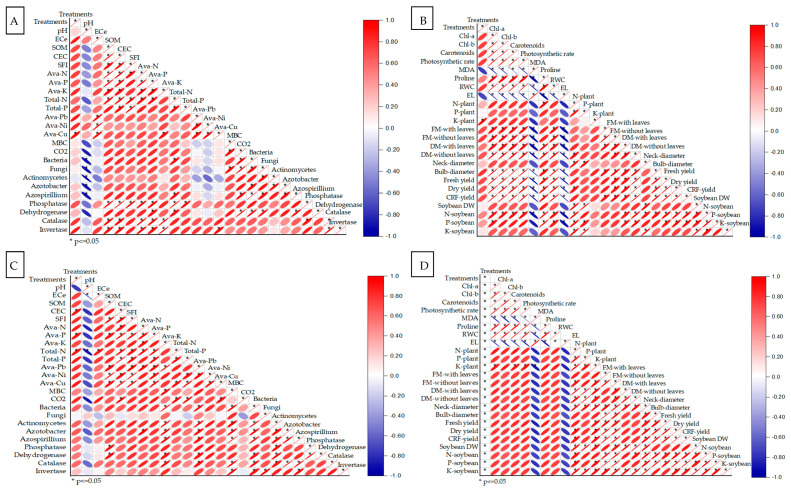
Pearson’s correlation matrix: (**A**) onion-soil properties, (**B**) onion-plant properties, (**C**) garlic-soil properties, and (**D**) garlic-plant properties, of soil and plant parameters of the onion (*Allium cepa* L.) and garlic (*Allium sativum* L.) plants grown in salt-affected soil and treated with the following treatments are: (i) T1: Control (no fertilizers or amendments), (ii) T2: Biofertilizer inoculation (*Azotobacter chroococcum* SARS 10 and *Azospirillum lipoferum* SP2) + recommended inorganic P and K, (iii) T3: 50% inorganic N and 50% organic manure (sewage sludge and poultry manure in a 1:1 ratio based on N content) + recommended P and K, (iv) T4: Biofertilizer + T3, and (v) T5: Conventional inorganic NPK fertilizers. The recommended inorganic NPK was calculated per hectare for each crop according to the recommendations from the Egyptian Ministry of Agriculture and Land Reclamation. Data are significant according to Tukey’s test at *p* < 0.05.

The correlation matrix from the field experiment on onion and garlic plants in salt-affected soil reveals significant relationships between soil and plant parameters across treatments, with similar trends observed for both crops (Figure 7). Soil pH showed a strong positive correlation with ECe (0.8) and SOM (0.6), indicating that higher pH levels are linked to increased salinity and organic matter, while CEC correlated positively with the SFI (0.6) and Ava-N (0.5), suggesting improved soil fertility. Biofertilizer treatments were strongly correlated with Total-N (0.7–0.8) and Total-P (0.5–0.6), reflecting enhanced nutrient availability, and Ava-Pb and Ava-Ni showed positive associations with microbial activity, including bacteria (0.6) and fungi (0.4). Enzyme activities, such as phosphatase (0.8) and dehydrogenase (0.6), were strongly correlated with Ava-Cu and MBC, indicating improved soil health with biofertilizer and organic treatments. For plant parameters, Chl a and Chl b had strong positive correlations with photosynthetic rate (0.8) and carotenoid content (0.6), showing that nutrient amendments enhanced photosynthetic efficiency, while plant DM and FM with leaves correlated positively with plant nitrogen (N-plant, 0.7) and potassium (K-plant, 0.6). Bulb diameter and fresh yield showed strong positive correlations with soybean DW (0.7) and CRF-yield (0.6), with T4 (biofertilizer combined with organic manure) and T5 (conventional NPK fertilizers) yielding the highest values, highlighting the effectiveness of integrated nutrient management in saline conditions.

**Figure 8:**
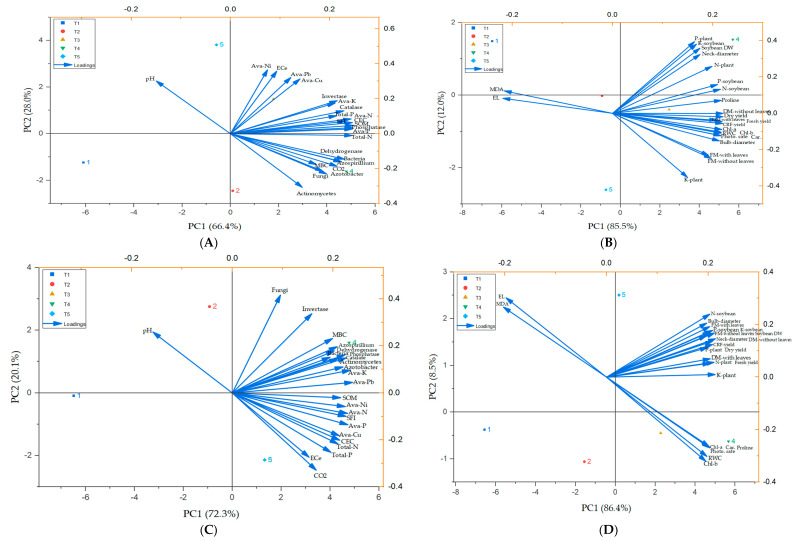
Principal component analysis: (**A**) onion-soil properties, (**B**) onion-plant properties, (**C**) garlic-soil properties, and (**D**) garlic-plant properties, of soil and plant parameters of the onion (*Allium cepa* L.) and garlic (*Allium sativum* L.) plants grown in salt-affected soil and treated with the following treatments are: (i) T1: Control (no fertilizers or amendments), (ii) T2: Biofertilizer inoculation (*Azotobacter chroococcum* SARS 10 and *Azospirillum lipoferum* SP2) + recommended inorganic P and K, (iii) T3: 50% inorganic N and 50% organic manure (sewage sludge and poultry manure in a 1:1 ratio based on N content) + recommended P and K, (iv) T4: Biofertilizer + T3, and (v) T5: Conventional inorganic NPK fertilizers. The recommended inorganic NPK was calculated per hectare for each crop according to the recommendations from the Egyptian Ministry of Agriculture and Land Reclamation.

Principal Component Analysis (PCA) of the field experiment on onion and garlic plants in salt-affected soil reveals distinct groupings of soil and plant parameters across treatments, highlighting the impact of integrated nutrient management (Figure 8). For onion, PC1 (49.5%) and PC2 (40.5%) explain most variability. Onion samples cluster positively along PC2, linked to soil parameters like dehydrogenase, MBC, catalase, fungi, *Azospirillum*, and *Azotobacter*, indicating enhanced microbial activity and soil health. Conversely, they group negatively along PC1, associated with plant parameters such as bulb diameter, fresh yield, soybean DW, P-soybean, N-soybean, and photosynthetic rate, reflecting improved growth and productivity. For garlic, PC1 (86.36%) and PC2 (4.48%) account for the variability. Treatments T1 (control) and T2 (biofertilizer + phosphorus and potassium) cluster near the origin, indicating baseline conditions with minimal changes. T3 (50% inorganic nitrogen + organic manure) and T5 (conventional NPK) show a negative shift along PC1, linked to stress indicators like EL and MDA. In contrast, T4 (biofertilizer + T3) shifts positively along PC1, strongly correlated with beneficial plant parameters (fresh yield, bulb diameter, dry mass with leaves, photosynthetic rate) and nutrient contents (N-plant, P-plant, K-plant). Soil parameters show pH negatively correlated with PC1, while soil organic matter (SOM), dehydrogenase, MBC, fungi, *Azospirillum*, *Azotobacter*, and enzyme activities (phosphatase, catalase, invertase) are positively correlated, indicating improved soil health and microbial activity. Overall, T4 and T5 demonstrate the strongest positive effects on soil and plant performance, underscoring the effectiveness of combining biofertilizers with organic and inorganic amendments in saline conditions.

## 3. Discussion

### 3.1. Impact of Nitrogen Amendments on Soil Properties and Fertility

The results of this study demonstrate that nitrogen amendments, particularly the combination of biofertilizers and organic–inorganic fertilizers (T4), significantly improved soil fertility and nutrient availability in salt-affected soils. The increase in SOM and CEC under T4 (24.7 for onion and 24.8 for garlic) compared to the control (T1) highlights the role of organic amendments in enhancing soil structure and nutrient retention [23]. This is consistent with previous studies that have shown that organic manure improves soil aggregation and water-holding capacity, which are critical in saline soils [26]. The elevated levels of Ava-N, Ava-P, and Ava-K in T4 (37.4–39.7 mg/kg for onion and 35.4–35.7 mg/kg for garlic) further support the effectiveness of integrated fertilization strategies in mitigating nutrient deficiencies in salt-affected soils [27].

The increase in soil salinity (EC_e_) with organic and inorganic amendments, particularly in T4 (4.99 for garlic), suggests that while these treatments improve fertility, they may also contribute to salt accumulation. However, the enhanced SOM and microbial activity under T4 likely offset the negative effects of salinity by improving soil structure and nutrient cycling [18]. This is in line with findings by [5], who reported that organic amendments can buffer the adverse effects of salinity by promoting microbial activity and nutrient availability. The increase in soil fertility indicators such as SOM and CEC under T4 is particularly significant because these properties are often degraded in saline soils due to the displacement of calcium and magnesium by sodium ions, which disrupts soil structure [1]. The improvement in these properties under T4 suggests that the combination of biofertilizers and organic amendments can restore soil health even under high salinity conditions.

### 3.2. Microbial Responses to Nitrogen Amendments

The microbial community responded positively to biofertilizer and organic amendments, with T4 showing the highest bacterial (2.54 × 10^6^ CFU/g for onion) and fungal (2.9 × 10^6^ CFU/g for onion) populations. This is consistent with studies by [13], who found that biofertilizers enhance microbial diversity and activity, particularly in saline soils. The increased MBC and CO_2_ under T4 further indicate that biofertilizers and organic amendments stimulate microbial activity, which is crucial for nutrient mineralization and soil health [11]. The suppression of fungal populations under conventional inorganic fertilizers (T5) aligns with findings by [1], who noted that excessive use of chemical fertilizers can disrupt microbial balance. In contrast, the combination of biofertilizers and organic amendments in T4 promoted beneficial microbes such as *Azotobacter* and *Azospirillum*, which are known for their nitrogen-fixing capabilities and ability to enhance plant growth under stress conditions [24]. The role of microbial activity in improving soil fertility under saline conditions cannot be overstated. Microbes play a critical role in nutrient cycling, particularly in the mineralization of organic matter into plant-available forms of Ava-N, Ava-P, and Ava-K [18]. The increased microbial activity under T4 likely contributed to the higher levels of available nutrients observed in this treatment. Additionally, the presence of beneficial microbes such as *Azotobacter* and *Azospirillum* can enhance plant growth by producing growth-promoting substances such as auxins and cytokinins, which help plants cope with stress [13]. This is particularly important in saline soils, where plants often experience nutrient imbalances and oxidative stress.

### 3.3. Microbial Responses to Nitrogen Amendments

Soil enzymes such as dehydrogenase, phosphatase, catalase, and invertase serve as key indicators of microbial activity, nutrient cycling, and oxidative stress management in saline soils. Dehydrogenase, a marker of overall microbial respiration and activity, was significantly enhanced in T4 (1.03 mg TPF/kg soil/day for onion, 3.73 for garlic), reflecting improved microbial metabolism due to the synergistic effects of biofertilizers and organic amendments [28]. This increase (25–35%) aligns with higher microbial biomass carbon (30–40% in T4; Section 2.4), suggesting that *Azotobacter* and *Azospirillum* stimulated microbial communities, counteracting salinity suppression [21]. Phosphatase, involved in phosphorus solubilization, was also highest in T4 (58.8 mg p-nitrophenol/g soil/h for onion, 56.6 for garlic), indicating enhanced P availability (17.9 mg/kg for onion in T4) [29]. This enzyme’s elevation supports nutrient cycling in saline soils, where high Na^+^ limits P uptake, and organic manure provided substrates for microbial phosphate release [14]. Catalase mitigates oxidative stress by decomposing hydrogen peroxide (H_2_O_2_), a ROS elevated under salinity (initial EC_e_: 3.47 dS/m) [30]. Initial catalase activity (187 ± 13 µmole H_2_O_2_/g soil/15 min; Table 3) suggests baseline ROS management, likely enhanced in T4 by increased microbial populations and SOM (8–12% increase), as biofertilizers promote antioxidant enzyme activity [3,7]. Invertase facilitates carbon cycling by hydrolyzing sucrose, providing energy for microbes [31]. Initial invertase (5.78 ± 0.11 µmole glucose/g soil/day; Table 3) indicates moderate carbon metabolism, probably boosted in T4 due to higher SOM from organic amendments, supporting microbial energy needs in saline conditions [4,5]. While post-treatment data for catalase and invertase were not measured, their enhancement in T4 is inferred from improved soil health. Future studies should quantify all enzymes to elucidate their roles in salinity mitigation.

### 3.4. Enhancement of Photosynthetic Efficiency and Stress Tolerance

The significant increase in chlorophyll content (Chl a, Chl b, and carotenoids) and photosynthetic rate under T4 (2.5 mg/g for onion and 1.9 mg/g for garlic) highlights the role of biofertilizers and organic amendments in improving photosynthetic efficiency. This is consistent with findings by [22], who reported that organic amendments enhance chlorophyll synthesis and photosynthetic activity in salt-stressed plants. The reduction in MDA levels under T4 (9 nmol/g for onion and 15 nmol/g for garlic) indicates reduced oxidative stress, which is critical for maintaining membrane integrity and cellular function under salinity [19]. The accumulation of proline, an osmoprotectant, under T4 (8 µmol/g for garlic) further supports the role of biofertilizers and organic amendments in enhancing stress tolerance. This aligns with studies by [3], who found that proline accumulation is a key mechanism for osmotic adjustment in salt-stressed plants. The improved RWC under T4 (80% for garlic) also suggests that these treatments enhance water retention and reduce electrolyte leakage, thereby improving plant hydration and stress resilience [12]. The reduction in oxidative stress and improvement in water retention under T4 are particularly important because salinity often leads to the accumulation of ROS, which can damage cellular components such as lipids, proteins, and DNA [1]. By reducing oxidative stress and improving water retention, T4 helps plants maintain cellular function and growth under saline conditions.

### 3.5. Nutrient Uptake and Yield Enhancement

Soil salinity negatively impacts onion and garlic growth by disrupting nutrient uptake, reducing photosynthetic efficiency, and inducing oxidative stress. The control treatment (T1) exhibited the lowest yield and nutrient uptake, highlighting the detrimental effects of salinity on crop performance. However, the application of biofertilizers and organic amendments (T4) mitigated these effects by improving soil fertility, enhancing microbial activity, and promoting stress tolerance mechanisms. This aligns with findings by [1], who emphasized the importance of integrated soil management strategies in overcoming salinity-induced limitations.

Salinity affects plant growth through several mechanisms, including osmotic stress, ion toxicity, and nutrient imbalances [1]. Osmotic stress occurs when high salt concentrations in the soil reduce the availability of water to plants, leading to dehydration and reduced growth. Ion toxicity occurs when excessive sodium and chloride ions accumulate in plant tissues, disrupting cellular function and leading to oxidative stress. Nutrient imbalances occur when high sodium levels interfere with the uptake of essential nutrients such as potassium and calcium, which are critical for plant growth and stress tolerance [1]. The integrated fertilization strategy used in this study (T4) addresses these challenges by improving soil structure, enhancing nutrient availability, and promoting stress tolerance mechanisms such as proline accumulation and reduced oxidative damage.

The highest N, P, and K uptake under T4 (14.6 for garlic and 10.97 for onion) demonstrates the effectiveness of integrated fertilization strategies in enhancing nutrient availability and uptake. This is consistent with findings by [32], who reported that biofertilizers and organic amendments improve nutrient use efficiency in saline soils. The increased yield components, such as bulb diameter (5.2 cm for onion) and fresh yield (12.17 tons/ha for garlic), under T4 further highlight the positive impact of these treatments on crop productivity.

The mechanisms underlying these improvements include enhanced microbial activity, improved nutrient cycling, and increased stress tolerance through proline accumulation and reduced oxidative damage. These findings have significant implications for sustainable agriculture in salt-affected regions, offering a viable strategy for improving soil health and crop productivity without relying solely on chemical fertilizers [25]. The use of biofertilizers and organic amendments also aligns with the principles of sustainable agriculture by reducing the environmental impact of chemical fertilizers and promoting soil health [26].

### 3.6. Residual Impact of Nitrogen Amendments on Soybean Growth: Insights from the Bioassay Experiment

The bioassay experiment evaluating the residual effects of nitrogen amendments on soybean growth revealed significant improvements in soil nutrient availability and crop performance, particularly in plots treated with T4 (biofertilizer + 50% inorganic N and 50% organic manure). The highest soybean dry weight was observed in T4-treated plots (0.200 g for garlic and 0.194 g for onion), indicating sustained soil fertility that supported enhanced crop growth [33]. This aligns with findings by Ncayiyana et al. [32], who reported that organic amendments improve nutrient availability over multiple growing seasons, benefiting subsequent crops. The elevated N, P, and K content in soybean plants from T4-treated plots (73.1 mg/kg N, 927 mg/kg P, and 279 mg/kg K for garlic plots) underscores the effectiveness of integrated fertilization in enhancing nutrient availability and uptake efficiency [32]. Notably, garlic plots exhibited higher P content in soybean compared to onion plots, suggesting crop-specific residual effects on nutrient dynamics. Treatment T3 (50% inorganic N + 50% organic manure) also improved soybean dry weight and nutrient content compared to the control (T1), though it was less effective than T4, while conventional inorganic fertilizers (T5) showed moderate results. The control treatment (T1) consistently exhibited the lowest values for dry weight and nutrient content, emphasizing the necessity of fertilization strategies to maintain soil fertility in salt-affected soils. These findings highlight the long-term benefits of combining biofertilizers with organic amendments for sustaining nutrient availability and supporting crop productivity in rotation systems under saline conditions [26]. The sustained nutrient release from organic amendments, such as sewage sludge and poultry manure, likely contributed to the improved performance of soybean, offering a sustainable approach to enhancing soil fertility and crop yields in salt-affected regions [32].

### 3.7. Implications for Sustainable Agriculture

The findings of this study have significant implications for sustainable agriculture in salt-affected regions. The use of biofertilizers and organic amendments offers a viable alternative to conventional chemical fertilizers, which can have negative environmental impacts such as soil degradation, water pollution, and greenhouse gas emissions [26]. By improving soil health and crop productivity, these treatments can help farmers achieve sustainable yields while reducing their environmental footprint. The long-term benefits of biofertilizers and organic amendments are particularly important in the context of climate change, which is expected to increase the extent and severity of soil salinity in many regions [1]. By improving soil structure and fertility, these treatments can help build resilience to climate change and ensure food security in vulnerable regions. Additionally, the use of biofertilizers and organic amendments can contribute to the conservation of biodiversity by promoting the growth of beneficial microbes and reducing the need for chemical inputs [13].

## 4. Materials and Methods

### 4.1. Study Location and Experimental Setup

Field trials were carried out in Kafr El-Sheikh Governorate, Egypt, to assess the effects and residual impacts of different agronomic practices on soil health, plant physiology, and the productivity of onion (*Allium cepa* L.) and garlic (*Allium sativum* L.) cultivated in salt-affected soils. Soil samples (0–20 cm depth) were collected after the harvest of the preceding cotton crop. These samples were air-dried, sieved through a 2 mm mesh, and analyzed for key physicochemical and microbiological properties using established protocols [34]. The detailed characteristics of the experimental clay soil are provided in Table 3.

**Table 3:** Physical, chemical, and microbiological traits of experimental soil, sewage sludge, and poultry manure.

Parameter	Soil	Sewage Sludge	Poultry Manure
pH *	7.88 ± 0.01	5.54 ± 0.02	7.30 ± 0.01
Electrical conductivity (EC_e_; soil paste extract at 25 °C; dS/m)	3.47 ± 0.05	7.27 ± 0.08	9.24 ± 0.08
Saturation percentage (%)	77 ± 1	164 ± 2	125 ± 2
Total organic carbon (TOC; %)	1.29 ± 0.03	8.38 ± 0.05	9.35 ± 0.04
Soil organic matter (SOM; g kg^−1^)	22.2 ± 0.2	144.4 ± 1.4	161.2 ± 1.6
C/N ratio	13.16 ± 0.5	6.27 ± 0.3	7.14 ± 0.4
Cation exchange capacity (CEC; cmole_c_/kg soil)	33.9 ± 0.4	36.3 ± 0.5	na
Available-N (mg/kg)	42 ± 2.1	532 ± 22.2	490 ± 19.6
Available-P (mg/kg)	7 ± 0.05	54 ± 0.9	151 ± 2.4
Available-K (mg/kg)	103 ± 1.9	66 ± 0.9	515 ± 2.5
Total-N (g/kg)	0.98 ± 0.04	13.37 ± 0.81	13.09 ± 0.85
Total-P (mg/kg)	133 ± 21	444 ± 24	833 ± 28
DTPA-Cu (mg/kg)	0.92 ± 0.03	24.80 ± 0.91	4.90 ± 0.15
DTPA-Ni (mg/kg)	0.20 ± 0.01	2.80 ± 0.05	2.80 ± 0.03
DTPA-Pb (mg/kg)	0.70 ± 0.02	14.00 ± 0.08	3.60 ± 0.05
Sand (%)	18.20 ± 0.23	na ^†^	na
Silt (%)	57.54 ± 1.21	na	na
Clay (%)	24.26 ± 0.98	na	na
Texture class	Silty loam	na	na
Total bacterial count (CFU ^⸸^ 10^6^/g soil)	50.0 ± 1.21	133.0 ± 2.33	3.0 ± 0.02
Total fungi count (CFU 10^6^/g soil)	1.6 ± 0.01	5.0 ± 0.02	0.9 ± 0.01
Total actinomycetes count (CFU 10^6^/g soil)	3.3 ± 0.1	10.6 ± 0.3	1.8 ± 0.1
Spore-forming bacteria count (CFU 10^6^/g soil)	0.5 ± 0.01	0.6 ± 0.01	0.2 ± 0.01
*Azotobacter* count (CFU 10^4^/g soil)	8.8 ± 0.14	nd ^‡^	nd
*Azospirillium* count (CFU 10^4^/g soil)	2.1 ± 0.10	nd	nd
Phosphatase activity (mg p-nitrophenol/g soil/h)	35.5 ± 0.21	nd	nd
Dehydrogenase activity (mg TPF/kg soil/day)	6.91 ± 0.18	nd	nd
Catalase activity (µmole H_2_O_2_/g soil/15 min)	187 ± 13	nd	nd
Invertase activity (µmole glucose/g soil/day)	5.78 ± 0.11	nd	nd

* pH was measured in (1:2.5 *w*/*v* soil:water suspension and 1:10 biosolids:water suspension); ^†^ not available; ^‡^ not detected; ^⸸^ cell forming unit.

### 4.2. Source of Used Materials and Their Preparation

Sewage sludge was obtained from a nearby wastewater treatment facility, while poultry manure was sourced from a private broiler farm. Both materials were air-dried, ground, and sieved (2 mm) before being analyzed for their chemical and microbiological attributes using standard methods [35,36]. The properties of these organic amendments are summarized in Table 3.

The biofertilizer was formulated using two strains of plant-growth-promoting rhizobacteria (PGPR): *Azotobacter chroococcum* SARS 10 and *Azospirillum lipoferum* SP2, combined in a 1:1 ratio. These bacterial strains were provided by the Agricultural Microbiology Department of the Soils, Water, and Environment Research Institute (SWERI), Agricultural Research Center (ARC), Egypt. The inoculum was prepared according to the methods described by [37,38].

Onion (*Allium cepa* L.) seedlings of the cv. Tantawy and garlic (*Allium sativum* L.) bulbs were procured from a certified supplier in Kafr El-Sheikh Governorate, Egypt.

Nitrogen (N) was applied in the form of ammonium nitrate (33.5% N), phosphorus (P) as calcium superphosphate (15.5% P_2_O_5_), and potassium (K) as potassium sulfate (50% K_2_SO_4_). The application rates and methods followed the guidelines outlined by [34].

### 4.3. Experimental Treatments

The study evaluated five distinct treatments applied to onion and garlic crops: (T1) Control—No fertilizers or amendments were applied, (T2) Biofertilizer + inorganic P and K—This treatment involved inoculation with *Azotobacter chroococcum* SARS 10 and *Azospirillum lipoferum* SP2, combined with the recommended doses of inorganic phosphorus (P) and potassium (K), (T3) Combined organic and inorganic fertilization—This treatment consisted of 50% inorganic nitrogen (N) and 50% organic manure (a 1:1 mixture of sewage sludge and poultry manure based on N content), along with the recommended P and K, (T4) Biofertilizer + combined fertilization—This treatment combined biofertilizer inoculation with the fertilization regime of T3, and (T5) Conventional inorganic NPK—This treatment involved the application of standard inorganic NPK fertilizers.

The recommended rates of inorganic NPK fertilizers were determined per hectare for each crop (onion and garlic) based on guidelines from the Egyptian Ministry of Agriculture and Soil Reclamation. The treatments were tailored to the specific nutrient requirements and growth stages of onion and garlic grown in salt-affected soils.

#### 4.3.1. Onion Cultivation Treatments

T1: no fertilizers or amendments were applied, T2: applied 150 kg/ha of P_2_O_5_ during soil preparation, 115 kg/ha of K_2_SO_4_ at 60 days after sowing (DAS), and 3 L of biofertilizer at seedling transplantation, T3: applied the same P_2_O_5_ and K_2_SO_4_ rates as T2, with 160 kg/ha of N split into two equal applications (80 kg at 30 DAS and 80 kg at 60 DAS), T4: included the same P_2_O_5_, K_2_SO_4_, and N applications as T3, with the addition of 6000 kg/ha of sewage sludge and poultry manure during soil preparation, and T5: doubled the N application to 320 kg/ha (split into 160 kg at 30 DAS and 160 kg at 60 DAS) but excluded organic amendments.

#### 4.3.2. Garlic Cultivation Treatments

T1: no fertilizers or amendments were applied, T2: applied 180 kg/ha of P_2_O_5_ (distributed as 45 kg during soil preparation, 55 kg at germination, 55 kg at 30 DAS, and 25 kg at 60 DAS), 150 kg/ha of K_2_SO_4_ (25 kg at germination, 50 kg at 30 DAS, and 75 kg at 60 DAS), and 12 L of biofertilizer at bulb plantation, T3: applied the same P_2_O_5_ and K_2_SO_4_ rates as T2, with 127.5 kg/ha of N (47.5 kg at germination, 40 kg at 30 DAS, and 40 kg at 60 DAS), T4: included the same P_2_O_5_, K_2_SO_4_, and N applications as T3, with the addition of 8438 kg/ha of sewage sludge and poultry manure during soil preparation, and T5: applied the same P_2_O_5_ and K_2_SO_4_ rates as T4, with an increased N application of 255 kg/ha (95 kg at germination, 80 kg at 30 DAS, and 80 kg at 60 DAS), but excluded organic amendments.

### 4.4. Experimental Design and Crop Management

The experiments were arranged in a strip-plot design, with crops (onion and garlic) as row factors and treatments (T1–T5) as column factors. Each treatment was replicated three times. Individual plots measured 2 × 3 m, separated by 1 m wide ditches. Onion cultivation: Seedlings were transplanted at a spacing of 15 cm on ridges 100 cm wide, with a planting density of 357,000 seedlings per hectare. Garlic cultivation: Bulbs were sown at a spacing of 10 cm on ridges 60 cm wide, with a planting rate of 595 kg of bulbs per hectare.

Both crops were planted on 14 November and harvested on 25 April. Standard agronomic practices, including irrigation, weed control, and pest management, were followed according to national guidelines.

The microbial inoculum was prepared by incubating *Azotobacter chroococcum* SARS 10 and *Azospirillum lipoferum* SP2 for two days at 30 °C. A liquid inoculum with a density of 10^8^ CFU/mL was prepared. Onion seedlings and garlic bulbs were immersed in the inoculum at rates of 3 L and 12 L, respectively, prior to transplantation.

### 4.5. Soil Sampling and Analytical Procedures

Soil samples were collected at 80 days after sowing (DAS) from a depth of 0–20 cm in each experimental plot, with three replicates per treatment. Plant debris, stones, and gravel were carefully removed, and the samples were air-dried at room temperature. The samples were then ground, sieved through a 2 mm mesh, and stored in plastic bags for further analysis. Additional samples for biological and biochemical analyses were collected simultaneously, placed in sterilized polyethylene bags, and transported to the laboratory in an icebox. These samples were sieved through an 8 mm mesh and stored at −20 °C until analysis.

#### 4.5.1. Soil Physicochemical Analysis

Soil pH was measured in a 1:2.5 soil-to-water suspension using a pH meter (Genway 3510, Cambridgeshire, UK). EC_e_ was determined from soil paste extracts using an EC meter (Jenway 4310, Cambridgeshire, UK) as described by [34]. Soil organic matter (SOM) was quantified using the potassium dichromate oxidation method [34]. Cation Exchange Capacity (CEC) was evaluated using the sodium acetate saturation technique [34].

The soil fertility index SFI was calculated to assess the impact of fertilization treatments on soil health and productivity, following the methodology of [39]. The index incorporates key soil parameters such as pH, EC_e_, SOM, CEC, available nutrients (NPK), total nutrient content (NP), and trace metals (Cu, Ni, Pb).

$$SFI=\sum_{i=1}^{n}\left(\frac{observed\;value\;of\;soil\;parameter\;i}{Optimum\;value\;of\;soil\;parameter\;i}\right)\times weight\;of\;soil\;parameter\;i\;$$

where n is the total number of indicators, if no weights are given equal weights of parameters is assumed.

Available nitrogen (Ava-N) was extracted using 2M KCl and analyzed using the semi-micro Kjeldahl method [34]. Available phosphorus (Ava-P) was extracted using 0.5 M sodium bicarbonate [40] and quantified colorimetrically using the ascorbic acid method [34]. Available potassium (Ava-K) was extracted with ammonium acetate and measured using Atomic Absorption Spectrophotometry (AAS, PerkinElmer 3300, Shelton, CT, USA) [36]. Total nitrogen (Total-N) was determined in digested soil samples using a mixture of H_2_SO_4_ and HClO_4_ (10:3 *v*/*v*) and analyzed using the semi-micro Kjeldahl method [34]. Total phosphorus (Total-P) was measured in digested samples prepared with a mixture of HNO_3_ and HClO_4_ (10:3 *v*/*v*) and quantified using AAS (PerkinElmer 3300, Shelton, CT, USA) [36].

Extractable soil Cu, Pb, and Ni were extracted using DTPA (diethylene triamine penta acetic acid) and quantified with AAS (PerkinElmer 3300, Shelton, CT, USA) following [36].

#### 4.5.2. Microbiological Analysis

Microbial biomass carbon (MBC) was assessed using the chloroform fumigation-extraction procedure [41]. Carbon content in the extracts was determined using an Elementar Vario Max Cube analyzer (Elementar-Straße 1, 63505 Langenselbold, Hesse, Germany). Soil respiration was measured in thawed soil samples incubated at 35 °C for 10 days after the addition of 80 mg glucose/g soil. Respiration was determined over 24 h using NaOH to capture released CO_2_ [42].

Microbial populations were quantified using selective media: total bacterial count on Thornton’s medium, total fungal count on Martin’s medium, total actinomycetes count on Jensen’s medium, *Azotobacter* spp. on modified Ashby’s medium, and *Azospirillum* spp. on N-deficient semi-solid medium [43].

Dehydrogenase activity was determined spectrophotometrically using the 2,3,5-triphenyl tetrazolium chloride (TTC) method [28]. Phosphatase activity was measured using p-nitrophenyl phosphate as a substrate [29]. Catalase activity: was quantified using the titration method [44]. Invertase activity was evaluated using the Schaffer-Somogyi micro method [31].

### 4.6. Plant Sampling and Physiological Analysis

Seventy days after transplantation of seedlings (onion) and seeding of clove (garlic), the youngest fully expanded leaves were collected to assess physiological and biochemical stress markers.

RWC was measured using 1 cm^2^ leaf discs. Fresh mass (FM) was recorded, followed by submersion in distilled water for 5 h to determine turgid mass (TM). The discs were then dried at 70 °C for 48 h to obtain dry mass (DM). RWC was calculated using the formula by [45].

$$RWC\;\left(\%\right)=\frac{FM-DM\;}{TM-DM}\times 100$$

EL was determined using ten leaf discs incubated in distilled water at 55 °C for 25 min. Initial electrical conductivity (EC1) was measured, followed by heating to 100 °C for 10 min to measure EC2. EL was calculated as described by [46].

$$EL\;(\%)=\frac{EC1}{EC2}\times 100$$

MDA, a marker of lipid peroxidation, was measured using the thiobarbituric acid (TBA) method [47]. Absorbance was recorded at 532 nm and 600 nm using a UV-160A spectrophotometer (Shimadzu, Kyoto, Japan).

Proline was determined from fresh leaves ground in 3% H_2_SO_4_. The homogenate was centrifuged, and the supernatant was mixed with toluene. Proline concentration was quantified using ninhydrin reagent [48].

Plant tissues were digested in H_2_SO_4_, and nitrogen content was analyzed using the Kjeldahl method. Phosphorus and potassium were quantified using AAS (PerkinElmer 3300, Shelton, CT, USA).

Chlorophyll and carotenoid concentrations (µg/g) were determined using 80% acetone extracts [49]. Absorbance was measured at 645 nm, 663 nm, and 470 nm using a UV-160A spectrophotometer (Shimadzu, Kyoto, Japan).

Chlorophyll a = 12.7 (A_663_) − 2.69 (A_645_)

Chlorophyll b = 25.8 (A_645_) − 4.68 (A_663_)

Carotenoids = (1000 (A_470_) − 2.27 (chl a) − 81.4 (chl b))/227

Photosynthetic rate was measured using the LI-6400 portable photosynthesis system (Li-COR, Lincoln, NE, USA) under optimal conditions.

At harvest, ten plants per plot were randomly selected to measure fresh mass, dry mass, neck diameter, and bulb diameter. The fresh and dry yields were calculated based on the plot area.

The contribution rate of fertilizers (CRF; %) for onion and garlic yields was calculated to assess the effects of different fertilizer treatments on crop productivity using the formula [50]:

$$\mathrm{CRF}\;=\;\frac{\left(Y\;fertilizer-Y\;control\right)}{Y\;fertilizer}\times 100$$

where Y control is yield in the control plot (ton/ha) and Y fertilizer is yield in the fertilized plot (ton/ha).

### 4.7. Bioassay Trial

A bioassay trial was carried out to assess the residual impacts of the applied treatments (T1–T5). Soil samples (0–20 cm depth) were collected from the experimental plots at harvest and used to cultivate soybean (*Glycine max* L.) plants. Soybean was selected for the bioassay trial due to its well-documented sensitivity to soil nutrient availability, particularly nitrogen, phosphorus, and potassium, which are critical indicators of residual soil fertility following onion and garlic cultivation in salt-affected soils. In brief, test tubes measuring 30 cm in height and 4 cm in diameter were filled with 50 g of air-dried soil. Five soybean seeds were planted in each tube and covered with a thin layer (1–2 cm) of sterilized washed sand. The tubes were then irrigated to approximately field capacity using sterilized distilled water. Before sowing, the seeds were sterilized by immersing them in 50% H_2_SO_4_ for 2 min, followed by thorough rinsing with sterile distilled water until a neutral pH (7) was achieved. After one week, the seedlings were thinned to two plants per tube. The experiment was designed as a randomized complete block with nine replicates per treatment. Whole soybean plants were harvested 21 days after sowing, and fresh mass (FM), dry mass (DM), and NPK content were measured.

### 4.8. Statistical Analysis

The collected data were analyzed using analysis of variance (ANOVA) following the methodology described by [51]. Significant differences were identified using Tukey’s test at a significance level of *p* ≤ 0.05. A one-way ANOVA was conducted to evaluate the effects of treatments on the yield and yield components of onion and garlic separately. For other soil and plant parameters, a two-way ANOVA was performed to assess the influence of treatments and vegetative cover (onion and garlic).

## 5. Conclusions

This study demonstrates that integrated fertilization strategies, particularly the combination of biofertilizers and organic–inorganic amendments (T4), significantly improve soil fertility, enhance crop growth, and increase productivity in salt-affected soils. By increasing SOM, CEC, and nutrient availability, while promoting microbial activity and stress tolerance, these treatments offer a sustainable solution to the challenges posed by soil salinity. The findings highlight the potential of biofertilizers and organic amendments to reduce reliance on chemical fertilizers, improve soil health, and enhance crop yields, making them a viable option for sustainable agriculture in saline environments. Future research should focus on optimizing these treatments for different crops and soil types, exploring their long-term effects, and developing cost-effective application methods to support farmers in adopting these practices. This approach not only addresses immediate productivity challenges but also contributes to long-term soil resilience and food security in salt-affected regions.

## Figures and Tables

**Table 1:** Response of different soil properties to different nitrogen amendments (T1–T5) ^†^ and under onion (*Allium cepa* L.) and garlic (*Allium sativum* L.) cultivation in salt-affected soil.

	T1		T2		T3		T4		T5	
	Onion	Garlic	Onion	Garlic	Onion	Garlic	Onion	Garlic	Onion	Garlic
pH	8.20 ± 0.03 b	8.20 ± 0.02 a	7.90 ± 0.03 d	8.10 ± 0.03 b	8.10 ± 0.03 c	8.00 ± 0.03 c	7.90 ± 0.03 d	8.10 ± 0.03 b	8.30 ± 0.01 a	8.00 ± 0.02 c
EC_e_^‡^	4.00 ± 0.15 d	3.84 ± 0.05 c	4.06 ± 0.12 d	4.38 ± 0.10 b	4.49 ± 0.18 b	4.99 ± 0.10 a	4.27 ± 0.17 c	4.38 ± 0.07 b	4.71 ± 0.18 a	4.99 ± 0.10 a
SOM ^¥^ (%)	22.8 ± 0.40 d	22.2 ± 0.20 c	23.8 ± 0.41 c	22.4 ± 0.30 c	24.3 ± 0.40 b	23.4 ± 0.30 b	24.7 ± 0.41 a	24.8 ± 0.30 a	23.9 ± 0.20 c	23.4 ± 0.50 b
CEC ^⸸^	34.4 ± 0.50 d	34.1 ± 0.40 c	41.2 ± 0.53 c	36.1 ± 0.50 b	43.5 ± 0.59 b	46.2 ± 0.50 a	45.3 ± 0.65 a	46.7 ± 0.50 a	41.7 ± 0.28 c	46.2 ± 0.50 a
SFI *	0.94 ± 0.03 b	0.94 ± 0.02 b	0.99 ± 0.04 a	0.95 ± 0.02 b	0.99 ± 0.04 a	0.98 ± 0.02 a	1.00 ± 0.05 a	1.00 ± 0.03 a	0.99 ± 0.03 a	0.98 ± 0.02 a
Ava-N (mg/kg)	27.3 ± 1.32 e	28.0 ± 2.00 c	33.0 ± 1.34 d	33.0 ± 3.00 b	37.4 ± 1.56 b	35.4 ± 2.50 a	39.7 ± 1.56 a	35.7 ± 2.50 a	35.1 ± 0.72 c	35.4 ± 2.00 a
Ava-P (mg/kg)	8.5 ± 0.19 d	9.5 ± 0.30 d	12.3 ± 0.24 c	10.9 ± 0.40 c	15.4 ± 0.34 b	13.0 ± 0.30 b	17.9 ± 0.14 a	13.6 ± 0.50 a	12.9 ± 0.46 c	13.0 ± 0.30 b
Ava-K (mg/kg)	91 ± 1.02 c	95 ± 2.00 c	94 ± 1.24 c	103 ± 2.50 b	105 ± 1.05 b	103 ± 2.50 b	107 ± 1.36 a	108 ± 3.00 a	103 ± 2.82 b	103 ± 3.00 b
Total-N (g/kg)	1.23 ± 0.05 d	1.22 ± 0.02 b	1.44 ± 0.05 b	1.26 ± 0.03 b	1.45 ± 0.05 b	1.32 ± 0.03 a	1.51 ± 0.06 a	1.31 ± 0.04 a	1.39 ± 0.05 c	1.32 ± 0.02 a
Total-P (g/kg)	0.22 ± 0.01 b	0.25 ± 0.00 a	0.25 ± 0.01 a	0.25 ± 0.00 a	0.25 ± 0.00 a	0.26 ± 0.01 a	0.25 ± 0.00 a	0.26 ± 0.01 a	0.25 ± 0.00 a	0.26 ± 0.01 a
Ava-Pb (mg/kg)	0.61 ± 0.03 c	0.61 ± 0.02 c	0.65 ± 0.04 b	0.65 ± 0.02 b	0.69 ± 0.04 b	0.66 ± 0.02 b	0.66 ± 0.05 b	0.69 ± 0.03 a	0.72 ± 0.03 a	0.66 ± 0.02 a
Ava-Ni (mg/kg)	0.39 ± 0.02 d	0.38 ± 0.01 c	0.38 ± 0.03 d	0.39 ± 0.01 c	0.45 ± 0.03 b	0.42 ± 0.01 b	0.42 ± 0.04 c	0.45 ± 0.02 a	0.48 ± 0.02 a	0.42 ± 0.01 b
Ava-Cu (mg/kg)	1.33 ± 0.11 c	1.33 ± 0.03 c	1.37 ± 0.12 c	1.37 ± 0.04 c	1.97 ± 0.13 a	1.48 ± 0.04 b	1.84 ± 0.15 b	1.97 ± 0.05 a	2.10 ± 0.11 a	1.84 ± 0.05 b

^†^ (i) T1: Control (no fertilizers or amendments), (ii) T2: Biofertilizer inoculation (*Azotobacter chroococcum* SARS 10 and *Azospirillum lipoferum* SP2) + recommended inorganic P and K, (iii) T3: 50% inorganic N and 50% organic manure (sewage sludge and poultry manure in a 1:1 ratio based on N content) + recommended P and K, (iv) T4: Biofertilizer + T3, and (v) T5: Conventional inorganic NPK fertilizers. The recommended inorganic NPK were calculated per hectare per each crop according to the recommendations from the Ministry of Agriculture and Soil Reclamation, Egypt. ^‡^ Electrical conductivity of soil solution (dS/m); ^¥^ Soil organic matter; ^⸸^ Cation exchange capacity of soil (cmolc/kg); * Soil fertility index. Means in the same row of the same crop plant followed by different letters are significant according to Tukey’s test at *p* ≤ 0.05. Data are means ± SD. (*n* = 3).

**Table 2:** Changes in yield and yield components of onion (*Allium cepa* L.) and garlic (*Allium sativum* L.) plants cultivated in salt-affected soil after five different nitrogen amendments (T1–T5) ^¥^.

Parameter	T1		T2		T3		T4		T5	
	Onion	Garlic	Onion	Garlic	Onion	Garlic	Onion	Garlic	Onion	Garlic
Fresh mass with leaves (g/plant)	51.4 ± 0.12 d	68.5 ± 23 c	99.5 ± 0.15 b	70.6 ± 32 c	101.6 ± 0.16 b	84.4 ± 33 b	105.3 ± 0.16 a	92.1 ± 35 a	97.9 ± 0.09 c	84.4 ± 25 b
Fresh mass without leaves (g/plant)	43.6 ± 0.12 d	46.5 ± 23d	68.8 ± 0.15 c	51.1 ± 32 c	74.5 ± 0.16 b	58.1 ± 33 b	76.3 ± 0.16 a	62.8 ± 35 a	72.7 ± 0.09 b	58.1 ± 25 b
Dry mass with leaves (g/plant)	7.9 ± 0.71 d	16.3 ± 20 c	10.5 ± 0.77 b	21.2 ± 30 b	10.5 ± 0.95 b	21.6 ± 32 b	11.3 ± 1.1 a	23.1 ± 29 a	9.7 ± 0.44 c	21.6 ± 27 b
Dry mass without leaves (g/plant)	5.3 ± 2.54 c	11.4 ± 2.1 c	6.1 ± 2.62 b	14.4 ± 1.9 b	6.7 ± 2.67 a	15.2 ± 1.8 a	7.2 ± 2.70 a	15.4 ± 1.5 a	6.2 ± 1.32 b	15.2 ± 1.1 a
Neck diameter (cm)	1.86 ± 0.25 c	0.77 ± 0.2 c	1.85 ± 0.15 c	0.84 ± 0.3 b	1.91 ± 0.16 b	0.89 ± 0.2 b	1.98 ± 0.17 a	0.93 ± 0.2 a	1.84 ± 0.08 c	0.89 ± 0.4 b
Bulb diameter (cm)	3.8 ± 0.78 d	4.4 ± 0.2 b	4.5 ± 0.85 c	4.4 ± 0.4 b	5.0 ± 0.90 a	4.8 ± 0.5 a	5.2 ± 1.0 a	5.0 ± 0.4 a	4.8 ± 0.44 b	4.8 ± 0.3 a
Fresh yield (ton/ha)	9.8 ± 1.63 d	10.7 ± 0.02 c	10.6 ± 1.68 c	10.8 ± 0.03 c	11.3 ± 1.70 b	11.3 ± 0.04 b	11.8 ± 1.74 a	12.2 ± 0.04 a	10.9 ± 0.84 c	11.3 ± 0.05 b
Dry yield (ton/ha)	8.8 ± 96 d	9.0 ± 0.03 d	9.8 ± 107 c	9.2 ± 0.01 c	10.5 ± 342 b	9.7 ± 0.02 b	11.1 ± 541 a	10.2 ± 0.02 a	9.9 ± 611 c	9.7 ± 0.01 b
CRF ^†^	nc ^‡^	-	9.7 ± 0.12 d	1.8 ± 1.8 c	15.9 ± 0.23 b	6.9 ± 2.2 b	20.1 ± 0.25 a	11.5 ± 2.3 a	11.2 ± 0.22 c	6.9 ± 2.1 b

^¥^ (i) T1: Control (no fertilizers or amendments), (ii) T2: Biofertilizer inoculation (*Azotobacter chroococcum* SARS 10 and *Azospirillum lipoferum* SP2) + recommended inorganic P and K, (iii) T3: 50% inorganic N and 50% organic manure (sewage sludge and poultry manure in a 1:1 ratio based on N content) + recommended P and K, (iv) T4: Biofertilizer + T3, and (v) T5: Conventional inorganic NPK fertilizers. The recommended inorganic NPK were calculated per hectare per each crop (onion and garlic) according to the recommendations from the Ministry of Agriculture and Soil Reclamation, Egypt. ^†^ Contribution rate of fertilizers; ^‡^ Not calculated. Means in the same row followed by different letters are significant according to Tukey’s test at *p* ≤ 0.05. Data are means ± SD. (*n* = 10).

## Data Availability

Data will be made available upon the request.
